# Next-generation CAR-T engineering for colorectal cancer: integrating targets, tumor microenvironment barriers, and emerging strategies

**DOI:** 10.3389/fimmu.2026.1829723

**Published:** 2026-06-23

**Authors:** Yajuan Yang, Zongyue Gao, Ping Song, Chao Wang, Kai Wang, Zuowu Xi, Ruirui Jing

**Affiliations:** 1 Henan Province Hospital of Traditional Chinese Medicine, The Second Affiliated Hospital of Henan University of Chinese Medicine, Zhengzhou, Henan, China; 2 Department of Surgical Oncology, Affiliated Hangzhou First People’s Hospital, Westlake University School of Medicine, Hangzhou, Zhejiang, China; 3 Department of Translational Medicine and Clinical Research, Sir Run Run Shaw Hospital, Zhejiang University School of Medicine, Hangzhou, Zhejiang, China

**Keywords:** antigen heterogeneity, CAR-T cell therapy, colorectal cancer, CRISPR/Cas9, next-generation engineering, tumor microenvironment

## Abstract

Chimeric antigen receptor (CAR)-T cell therapy for colorectal cancer (CRC) faces three major barriers: antigen heterogeneity, off−tumor toxicity, and the efficacy−safety trade−off. To overcome these obstacles, next−generation engineering strategies have emerged, including: (1) novel CRC−associated targets with improved tissue restriction; (2) architectural innovations such as armored CARs, optimized signaling (1XX, 28−ΔIL2RB−z(YXXQ)), and cytokine−arming; (3) combinatorial antigen−sensing circuits (AND/OR/NOT gates, SUPRA, synNotch); and (4) CRISPR−based editing for exhaustion−related knockouts (e.g., PD−1, Fas, TGFBR2) and site−specific CAR knock−in. Clinical evidence has demonstrated objective responses and disease stabilization in subsets of patients. However, the immunosuppressive tumor microenvironment—including inhibitory cells, dense stroma, and metabolic dysfunction—remains a critical hurdle limiting CAR−T persistence. Future directions should prioritize molecular typing−guided intervention, universal CAR−T, multicellular platforms (CAR−NK, CAR−M), and interdisciplinary collaboration. This review provides a framework for designing next−generation CAR−T therapies capable of achieving durable remissions in advanced CRC.

## 1 Introduction

**Figure 1:**
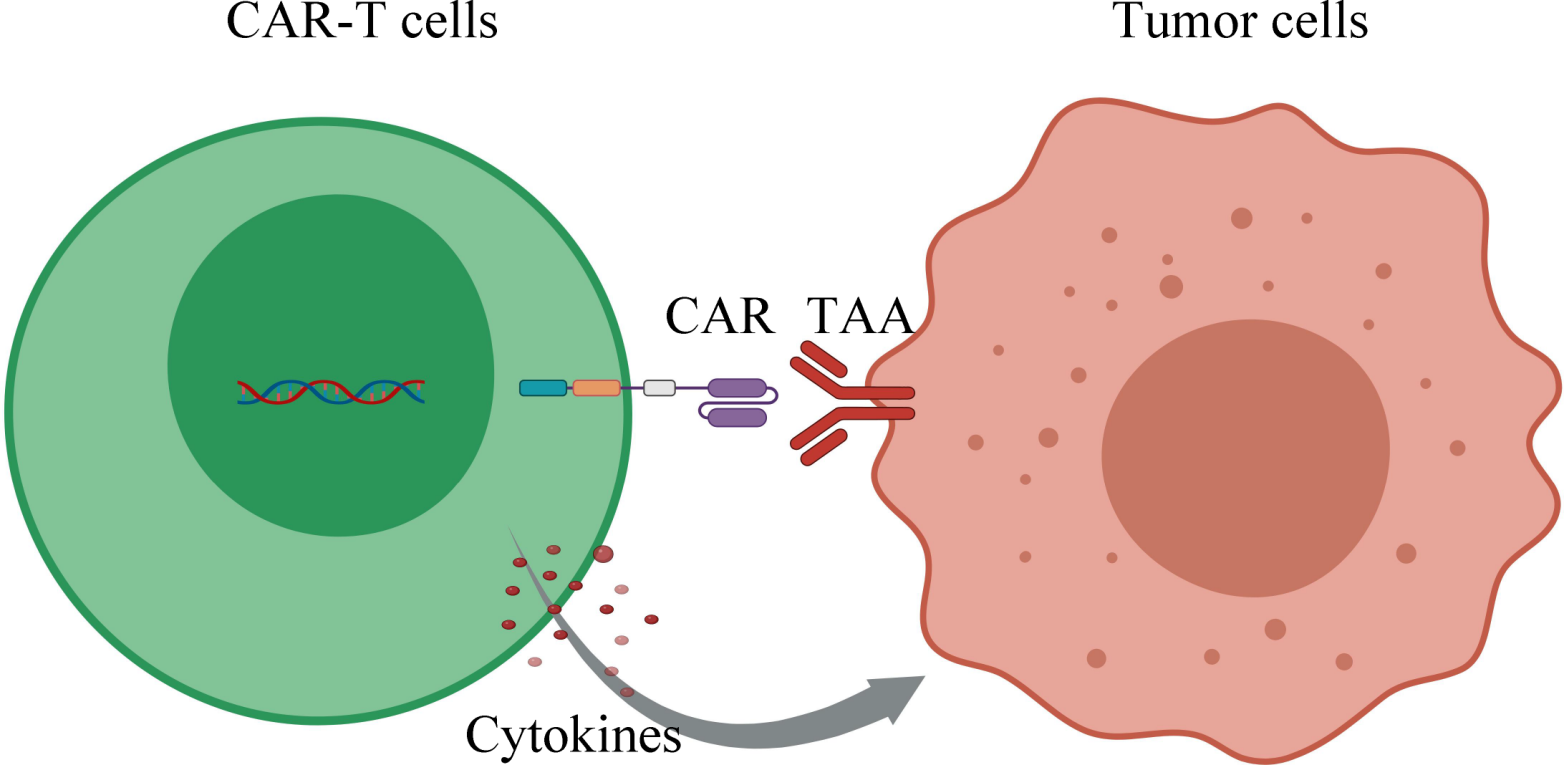
Schematic illustration of CAR-T cell recognition and killing of tumor cells.

Colorectal cancer (CRC) is the third most common and second deadliest cancer worldwide (1). Its pathogenesis involves interactions among genetics, lifestyle, and gut microbiota (2). While early-stage CRC is responsive to surgery and chemotherapy, the five−year survival rate for advanced-stage disease remains below 15% (3). Conventional therapies suffer from toxicity, resistance, and inability to address tumor heterogeneity (4). Immune cell therapy (e.g., CAR−T) has revolutionized hematological malignancies (5). The standard CAR-T manufacturing process involves isolating patient T cells, introducing CAR genes via viral or non−viral vectors, expanding the cells, and reinfusing them for targeted tumor killing (**Figure 1**).

Despite promising preclinical and early clinical data, most CAR−T strategies have failed to achieve durable remissions in solid tumors, including CRC. Three interrelated barriers consistently limit their translation. First, antigen heterogeneity allows outgrowth of antigen−negative clones after initial response, leading to relapse. Even for targets like CLDN18.2 (aberrant in ~35% of CRC), intratumoral heterogeneous expression inevitably causes treatment failure (6). AND−gate CARs miss single−antigen−positive cells, while OR−gates cannot cover complete antigen loss. Second, off−tumor toxicity remains a major hurdle (7, 8). CEA−targeted CAR−T causes dose−limiting colitis due to low−level CEA expression on normal colonic epithelium (1, 9). Newer targets such as GUCY2C and CDH17 offer improved tissue restriction (apical polarity or spatial masking) but lack clinical validation. NOT−gates (inhibitory CARs) require truly CRC−specific protective antigens, which are difficult to identify. Third, the efficacy−safety trade−off is particularly acute. Enhancing CAR−T potency-for example by disrupting PD−1 exacerbates off−tumor toxicity and cytokine release syndrome. Conversely, overly cautious designs (e.g., low−affinity CARs or complex logic gates) yield only disease stabilization rather than regression. Moreover, most current preclinical models poorly predict the spectrum of human toxicities, leading to frequent clinical failures.

The first three generations of CARs evolved from CD3ζ alone (first), to single co−stimulation (second), and then dual co−stimulation (third, more toxic). Subsequent next−generation designs have introduced features such as armored cytokine/suicide genes, gene editing, and enhanced STAT signaling, though no consensus definition exists for these newer platforms. This evolution highlights the ongoing challenge of balancing efficacy, specificity, and safety in CRC (10–12).

In this review, we first summarize the key barriers imposed by the CRC tumor microenvironment (TME) and tumor heterogeneity. We then discuss recent advances in next−generation CAR−T engineering, including the selection of novel targets, architectural engineering, combinatorial antigen−sensing circuits, and CRISPR−based modifications. Finally, we highlight the immunosuppressive TME as a critical clinical hurdle and propose future directions for translating these engineered cells into clinical applications.

While several excellent reviews have summarized CAR-T cell therapy for colorectal cancer (CRC), a critical gap remains: most existing syntheses do not holistically integrate three emerging pillars essential for overcoming the specific hurdles of microsatellite-stable (MSS) CRC-the predominant and immunologically “cold” subtype. First, logic-gated circuits (AND, OR, NOT, synNotch) enabling tumor-specific recognition while sparing normal tissue; second, CRISPR-based functional genomics as a tool not only for gene knockout but also for fine-tuning CAR signaling and overcoming epigenetic suppression unique to the CRC microenvironment; and third, a translational framework that directly maps engineering solutions to defined biophysical and immunological barriers (e.g., stromal fibrosis, TGF-β gradients, hypoxia). Unlike prior reviews that focus solely on engineering platforms or target lists, our work systematically links next−generation designs to CRC−specific barriers and functional themes, offering a clinically oriented resource. **Table 1** provides a comparative analysis with recently published reviews, highlighting our unique emphasis on logic-gated systems, CRC-specific TME barriers, CRISPR engineering strategies, and translational focus (**Table 1**) (13–15).

**Table 1:** Comparative analysis of this review with recent reviews on CAR-T therapy for colorectal cancer.

Feature/Aspect	Logic-gated circuits (AND/OR/NOT/synNotch)	CRC-specific TME barriers (CAFs, TGF-β, hypoxia, ECM stiffness)	CRISPR engineering beyond KO (CRISPRa/i, epigenome editing, multiplex knock-in)	Translational roadmap for MSS CRC	Comparison of engineering trade-offs (e.g., CD28 vs 4-1BB, viral vs non-viral)
This Review	Dedicated section (2.3) with clinical translation update	In-depth expansion (Section 4)	Comprehensive (Section 2.4)	Yes (Future perspectives with molecular typing)	Critical analysis throughout Sections 2.2 & 2.4
Review A (15)	Brief mention	General TME discussion	Mainly gene knockout	Brief mention	Sequential description
Review B (14)	Mention	General solid tumor barriers	Cas9 only for PD-1 KO	Partial	Sequential description
Review C (13)	Not covered	Focus on MSS immune evasion only	Not covered	Partial	Sequential description

## 2 Engineering strategies to enhance CAR−T therapy in CRC

**Figure 2:**
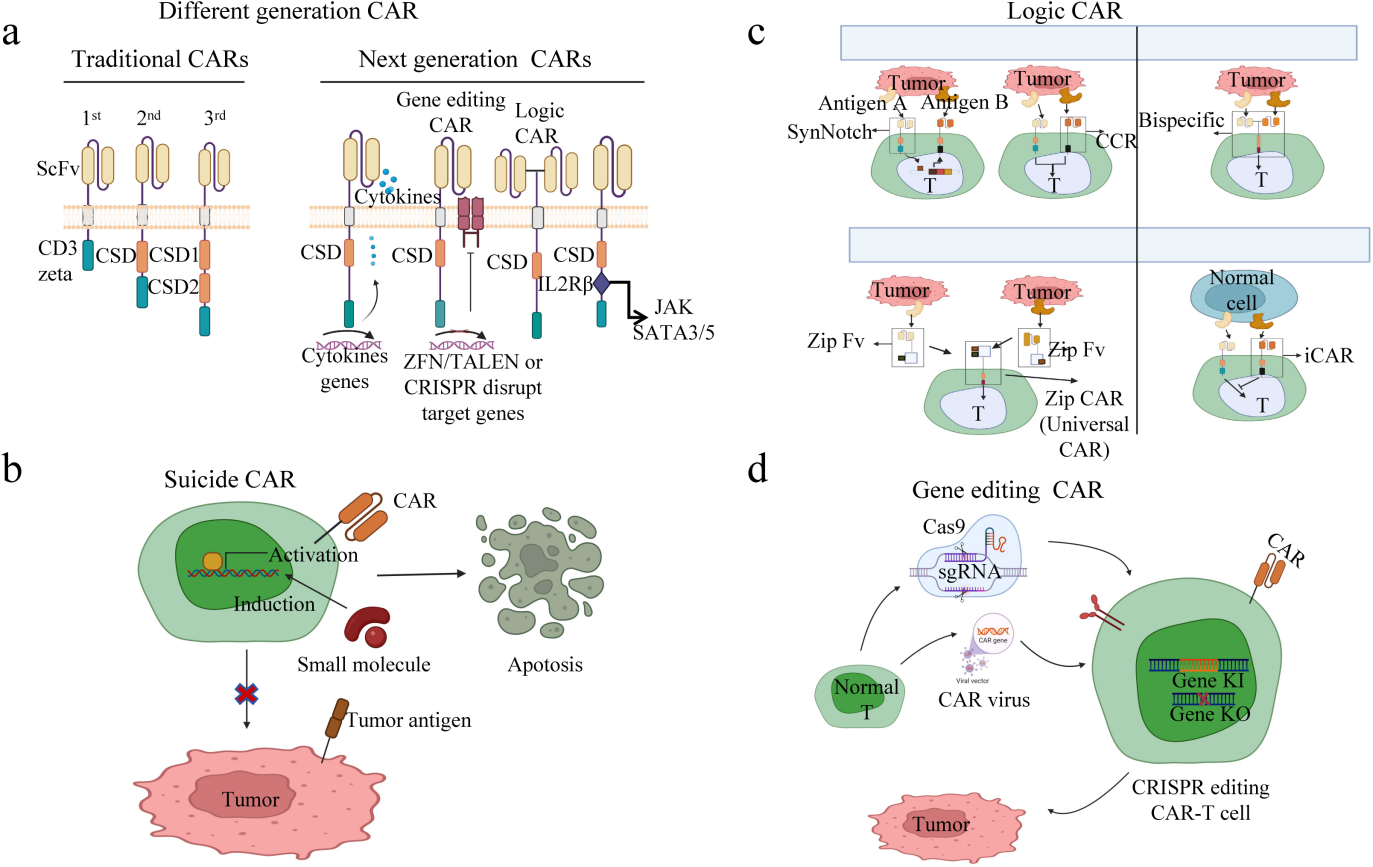
Schematic illustration of the development and engineering of CAR-T cells. **(a)** Structural evolution of CAR designs across generations. **(b)** Suicide CAR systems incorporating safety switches. **(c)** Logic-gated CAR strategies for enhanced tumor specificity. **(d)** CRISPR gene editing to generate functionally enhanced CAR-T cells. CSD, costimulatory domain.

Significant advances have been made in each area. First, we discuss emerging CRC−associated targets with improved tissue restriction and safety profiles. Second, we summarize architectural engineering strategies, including third−generation CARs as well as next−generation designs such as signaling−optimized constructs (e.g., 1XX CAR and 28−ΔIL2RB−z(YXXQ)) and cytokine−arming approaches that remodel the tumor microenvironment (16). Third, we describe combinatorial antigen−sensing circuits based on Boolean logic gates (AND, OR, NOT), SUPRA CARs, and synNotch receptors, which enhance tumor specificity and mitigate off−tumor toxicity (7). Finally, we examine CRISPR−based modifications-including knockout of inhibitory receptors and exhaustion−related molecules, as well as site−specific knock−in of CAR transgenes to generate safer, more potent universal CAR−T cells (**Figure 2**).

To address the major challenges of CRC CAR−T therapy-antigen targeting and escape, trafficking and infiltration, persistence and exhaustion, safety, and manufacturability—the field has developed four categories of engineering strategies. This section reviews these strategies in terms of: (1) novel CRC−associated targets; (2) architectural engineering; (3) combinatorial antigen−sensing circuits; and (4) CRISPR−based modifications. Within each category, we highlight how specific designs address one or more of these functional themes (**Table 2**).

**Table 2:** Functional classification of next−generation CAR−T engineering strategies for colorectal cancer.

Functional category	Key engineering strategies	Representative CRC−relevant examples (reference)
Antigen targeting & escape	Logic−gated circuits; dual−targeting	CEA/EpCAM AND gate (35); CEA−or−HER2 tandem CAR (7); HER2−synNotch/CEA−CAR (38); MSLN−targeted NOT−gate A2B694 (NCT06051695)
Trafficking & infiltration	Cytokine−armed CARs; vascular modulation	7X19 (IL−7+CCL19) CAR−T (29); IL−15/IL−21−armored GPC3 CAR (27, 28); IL−10−secreting CAR (30); IL−18−secreting DLL3 CAR (31); CA4P combination (59)
Persistence & exhaustion	Signaling−optimized; cytokine co−expression; CRISPR knockout of exhaustion genes	1XX CAR (32–34); 28−ΔIL2RB−z(YXXQ) CAR (13); PD−1−knockout CAR−T (39); RASA2−deficient CAR−T (47); MED12−knockout CAR−T (48); PRDM1−mutated CAR−T (50)
Safety control	NOT−gate (iCAR); AND−gate logic	CEA−activating CAR + VSIG2−iCAR; AND−gated RevCAR (35); A2B694 NOT−gate trial (NCT06051695)
Manufacturability & accessibility	Universal CAR−T (TCR/HLA knockout); Site−specific CAR knock−in; non−viral vectors	TRAC/PD−1 targeted CAR knock−in (53); pTRAC−CAR (54); TCR−/B2M−multiplex knockout UCAR−T (39)

### 2.1 Identification of novel CRC-associated targets

In recent years, research on CAR-T therapy for colorectal cancer (CRC) has gradually shifted from traditional broadly expressed targets to novel targets with higher tumor specificity or functional restrictions. CEA, as a classic CRC-associated antigen, is highly expressed in metastatic CRC and has accumulated substantial clinical data. However, its on-target/off-tumor toxicity on normal colonic epithelium (e.g., colitis) severely limits its application, and current dose-escalation phase I trials only achieve disease stabilization (9, 17). To reduce off-target risk, GUCY2C (GCC19) is considered to have low off-tumor toxicity due to its expression restricted to the intestinal epithelium with apical polarity, yet the intracellular epitope poses challenges for CAR design. Nonetheless, a phase I trial has reported promising efficacy with a 40% objective response rate (ORR), 6.0 months of progression-free survival (PFS), and 22.8 months of overall survival (OS) (18).

Another highly regarded target is CLDN18.2, which is aberrantly activated in approximately 35% of CRCs (particularly right-sided tumors and peritoneal metastases). Its normal expression is confined to the gastric mucosa, providing a good safety profile, but heterogeneous intratumoral expression remains a challenge. Representative strategies combine CLDN18.2-targeted CAR-T with PD-1 or TGF-β blockade (19, 20). In contrast, MUC1-STn is a tumor−specific aberrant O−glycosylation epitope of MUC1 in CRC, effectively avoiding the off−target toxicity associated with conventional targets and has achieved >80% tumor regression in PDX models (21).

CDH17 is a tight junction−associated protein that is spatially masked; this property spares normal cells from immune attack, enabling precise killing without autoimmune damage (22–24). For cancer stem cell (CSC)-targeted therapy, LGR5 and DCLK1 are enriched in intestinal stem cell−like and CSC populations, aiming to overcome tumor heterogeneity and recurrence. However, theoretical on−target toxicity to normal colonic stem cells exists, and strategies focus on eliminating the CSC pool to block recurrence (25, 26). Similarly, CD133, expressed on CSCs and chemoresistant subpopulations, has entered clinical trials. A phase I study reported 13% partial response (PR) and 60.9% stable disease (SD), along with self−limiting short−term hematologic toxicity (27).

In summary, the selection of CAR-T targets for CRC is evolving from highly expressed but poorly safe conventional antigens (e.g., CEA) toward molecules with strict tissue restriction, unique spatial epitopes, or tumor−specific glycosylation modifications (e.g., GUCY2C, CDH17, MUC1-STn), as well as cancer stem cell markers (LGR5, DCLK1, CD133) (**Table 3**). Together, these novel targets address the challenge of antigen targeting and safety by improving tumor specificity and reducing off-tumor toxicity.

**Table 3:** Summary of CAR-T target antigens in colorectal cancer.

Target antigen	Expression features in CRC	Key advantages	Main challenges/limitations	Representative CAR-T strategies
CEA (53, 54)	Highly expressed in metastatic CRC	Well−established clinical data	On−target/off−tumor toxicity in normal colon (colitis)	Dose−escalation phase I trial: disease stabilization
GUCY2C (GCC19) (55)	Restricted to intestinal epithelium; apical polarity	Low off−tumor risk	Intracellular epitope limits CAR design	Phase I trial: 40% ORR, PFS 6.0 mo, OS 22.8 mo
CLDN18.2 (36, 37)	Aberrantly activated in ~35% of CRC (right−sided, peritoneal metastases)	Normal expression confined to gastric mucosa; good safety profile	Heterogeneous expression within tumor	Combined with PD−1 or TGF−β blockade
MUC1-STn (38)	Tumor−specific aberrant O−glycosylation epitope of MUC1 in CRC	Avoids off−target toxicity of conventional targets	Not discussed in the reviewed studies	>80% tumor regression in PDX models
CDH17 (39–41)	Tight junction−associated protein; spatially masked	Spared access to normal cells; no autoimmune damage	Not discussed in the reviewed studies	Exploits spatial masking for precise killing
LGR5/DCLK1 (42, 43)	Enriched in intestinal stem cell−like and CSC populations	Targets tumor stemness and heterogeneity	Theoretical on−target toxicity to normal colonic stem cells	Eliminates CSC pool, blocks recurrence
CD133 (59)	Expressed on CSCs and chemoresistant subpopulations	Clinical data available; targets recurrence/resistance	Self−limiting short−term hematologic toxicity	Phase I trial: 13% PR, 60.9% SD

### 2.2 Architectural engineering of CAR-T cells

CAR−T cell therapy for colorectal cancer (CRC) has advanced rapidly through generational upgrades and strategic innovations (**Figure 2**). Third−generation CAR−T cells, incorporating dual co−stimulatory domains (e.g., CD28/4−1BB), have significantly improved anti−tumor efficacy and persistence in the complex tumor microenvironment. However, whether this benefit translates consistently to CRC remains debated, as some studies report increased toxicity without proportional gain in long−term tumor control. Further optimizations include structural domain engineering (e.g., replacing CD28 with the CD8α hinge to enhance antigen sensitivity and clearance of low−expressing tumors) (28), co−stimulatory synergy (e.g., combining CD27 with CD28/4−1BB to reduce exhaustion markers and promote memory T cells) (29), and non−viral vector construction. These strategies have enhanced CAR−T anti−tumor effects and mitigated potential immune escape mechanisms.

In the field of solid tumor therapy, next−generation “armored” CAR−T cells have emerged as a core innovation to overcome the limitations of conventional CAR−T approaches. These cells actively remodel the tumor microenvironment and recruit endogenous immune cells by secreting functional cytokines. Guided by this design concept, researchers have developed multiple strategies: GPC3−targeted CAR−T cells co−expressing IL−15 (designated 15.CAR) significantly enhanced *in vivo* expansion, intratumoral persistence, and anti−tumor activity, achieving an objective response rate of 33% (30). Furthermore, GPC3 CAR−T cells co−expressing both IL−15 and IL−21 (21.15.GBBz) maintained a stem cell/central memory phenotype via upregulation of TCF−1, leading to further improvements in expansion, persistence, and efficacy (31). 7X19 CAR−T cells, engineered to secrete IL−7 and CCL19, achieved superior infiltration and anti−tumor activity in liver and pancreatic cancer models, and facilitated complete tumor regression in patients with advanced disease (32). In addition, CAR−T cells secreting IL−10 induced a stem cell−like memory response in lymphoid organs, conferring durable protective immunity against tumor rechallenge (33); meanwhile, DLL3−targeted CAR−T cells secreting IL−18 demonstrated potent anti−tumor efficacy in a small cell lung cancer model by enhancing activation, alleviating exhaustion, inducing a memory phenotype, and remodeling the tumor immune microenvironment (34). A critical caveat is that most cytokine−arming studies compare against unarmed CARs rather than against optimized conventional CARs with superior costimulation, making it difficult to isolate the true benefit of cytokine secretion. Additionally, constitutive cytokine production carries systemic toxicity risks that have not been systematically evaluated in CRC models.

To address T cell activation−induced exhaustion, Michel Sadelain’s group mutated the ITAMs within the CD3ζ domain to encode a single immunoreceptor tyrosine−based activation motif (ITAM), generating the 1XX CAR. This design directs T cells toward a balanced effector and memory program, thereby conferring enhanced therapeutic properties. The 1XX CAR−T cell has demonstrated superior efficacy compared to conventional CAR−T cells in both hematological and solid tumors (35–37). A novel CAR, designated 28−ΔIL2RB−z(YXXQ), incorporates a truncated IL−2Rβ chain and a YXXQ motif, enabling antigen−dependent JAK/STAT signaling (16). This design promotes CAR−T cell proliferation and persistence while preventing terminal differentiation, resulting in superior antitumor effects against both liquid and solid tumors compared with conventional CAR constructs. Nevertheless, both designs have been validated predominantly in hematological or limited solid tumor settings; their efficacy against low−antigen−density CRC cells-which frequently downregulate surface targets-and within the highly fibrotic MSS CRC TME remains largely unexplored.

While the above studies collectively demonstrate impressive preclinical advances, it is important to recognize several unresolved trade-offs that limit direct translation to CRC patients. First, the choice of costimulatory domain (CD28 vs. 4−1BB vs. CD27) remains context−dependent. CD28 confers rapid and strong activation but accelerates exhaustion and terminal differentiation, whereas 4−1BB promotes sustained signaling and memory formation. For CRC, where tumors are often slow−growing yet embedded in a dense, suppressive stroma, the optimal balance is unclear, and third−generation CARs combining both domains have shown mixed results with increased toxicity in some reports. Second, as noted above, cytokine arming carries systemic toxicity risks that require careful evaluation in CRC-specific models. Third, the 1XX and 28−ΔIL2RB−z(YXXQ) signaling−optimized CARs need further validation in CRC. Finally, head−to−head comparisons of different architectural designs in physiologically relevant CRC models-such as patient−derived organoids or syngeneic immunocompetent MSS models are lacking. Future engineering should prioritize standardized benchmarking platforms that assess persistence, exhaustion resistance, and safety simultaneously, rather than sequential reporting of isolated improvements. Armored CARs and signaling−optimized designs primarily tackle trafficking, infiltration, persistence, and exhaustion.

### 2.3 Combinatorial antigen−sensing logic−gated CAR−T cells for colorectal cancer immunotherapy

In next−generation CAR−T engineering, dual−target Boolean logic gates have been introduced to enhance tumor specificity and mitigate off−tumor toxicity. An AND−gate design (e.g., CEA/EpCAM system) requires simultaneous engagement of two antigens for full activation, thereby limiting cytotoxicity to tumor cells co−expressing both markers (38).

Beyond the AND gate, other Boolean operations have been applied to CRC CAR−T cells. OR−gate CARs are activated upon recognition of any antigen within a defined panel, enabling broader coverage of heterogeneous tumors. For example, a tandem CAR targeting either CEA or HER2 effectively eliminated CRC cell pools expressing only one of the two antigens (7). NOT−gate (inhibitory CAR, iCAR) provides a safety override: recognition of a “protective” antigen on normal cells delivers an inhibitory signal that suppresses activation (39). In CRC models, an activating CAR against CEA combined with an iCAR against a colon−restricted antigen (e.g., VSIG2) prevented colitis. This NOT−gate logic has advanced to early clinical validation; the EVEREST−2 trial (NCT06051695) evaluates A2B694, a logic−gated Tmod™ CAR−T targeting mesothelin (MSLN) with an HLA−A*02 blocker, and has enrolled CRC patients.

SUPRA CARs (split, universal, programmable) integrate multiple logic operations into a single platform. The CAR is split into a universal ZipCAR and a ZipFv that assemble via leucine zippers, allowing AND, OR, or NOT logic to be reconfigured by swapping modules. In CRC cell lines, SUPRA CARs configured with AND logic required dual antigen binding, thereby reducing off−tumor lysis (40).

The synthetic Notch (synNotch) receptor implements a sequential AND gate. Upon binding a first antigen, synNotch releases a transcription factor that drives expression of a second CAR against a different antigen. For CRC, a HER2−synNotch/CEA−CAR system eliminated HER2−amplified tumors without attacking normal colonic cells with low HER2 expression—a key advantage over conventional anti−HER2 CAR−T cells (41). This design has also been extended to CAR−NK platforms for off−the−shelf CRC therapy. Despite their promise, most combinatorial logic circuits remain preclinical, with challenges related to vector packaging capacity and manufacturing complexity. Logic−gated circuits directly address antigen escape and safety.

### 2.4 CRISPR-based gene modification

Currently, most CAR−T cell manufacturing relies on autologous sources, which suffer from high cost and long production times. Using T cells from healthy donors to produce “off−the−shelf” CAR−T cells can improve accessibility, but graft−versus−host disease (GVHD) remains a major obstacle. Knockout of T−cell receptor (TCR)−related genes (e.g., TRAC, TRBC) and β2−microglobulin (B2M) to eliminate MHC class I expression via CRISPR/Cas9 significantly reduces alloreactivity. Multiplex−knockout CAR−T cells (e.g., targeting TRAC, B2M, and PDCD1) have demonstrated the potential to reduce GVHD while enhancing anti−tumor activity in preclinical studies (42).

CRISPR screens have identified multiple knockout targets that enhance CAR−T function. Inhibitory receptors (PD−1, CTLA4, TIGIT, LAG−3, CD244, CD160, TIM3) drive exhaustion; their knockout improves T cell activation. Additional beneficial knockouts include: Fas (improves AICD resistance and persistence) (43), LAG−3 (44), TGFBR2 (promotes central memory and solid tumor clearance) (45), adenosine A2A receptor (enhances HER2 CAR−T efficacy) (46), DGK (increases killing in glioma) (47), and GM−CSF (reduces CRS and improves anti−leukemia efficacy) (48, 49). Further screens revealed RASA2 (a RasGAP; ablation enhances MAPK signaling and cytolytic activity) (50), MED12, CCNC (inactivation increases expansion and metabolic fitness) (51), TLE4, IKZF2 (knockout enhances efficacy against glioblastoma) (52), and PRDM1 (mutation increases proliferation, stem−like properties, and *in vivo* efficacy (53). The cBAF complex was also identified as crucial for T cell fate decisions (54), and PRODH2 overexpression (gain−of−function) enhances CAR−T efficacy (55).

While this expanding list of beneficial knockouts is impressive, three critical caveats apply: (1) most screens were performed in leukemia models or standard culture, not in CRC−relevant immunosuppressive conditions (TGF−β−rich, hypoxic, adenosine−high); (2) multiplex editing-knocking out three or more genes-carries cumulative genotoxicity (chromosomal rearrangements, p53 activation) that remains poorly characterized in primary human T cells; (3) the optimal knockout combination likely depends on both the CAR architecture and the specific CRC TME barrier profile, but head−to−head comparisons are lacking.

Random integration by traditional retroviral/lentiviral vectors carries a risk of oncogenesis. Targeted knock−in of the CAR transgene at specific genomic loci (e.g., TRAC, PD−1) using CRISPR/Cas9 generates CAR−T cells with lower differentiation, reduced exhaustion, and superior anti−tumor efficacy (56). Non−viral, plasmid−based pTRAC−CAR−T cells offer the advantages of low cost and high safety (57). CRISPR−based modifications enhance persistence (exhaustion knockout), safety (suicide genes, UCAR−T to avoid GVHD), and manufacturability (off−the−shelf platforms).

Despite the compelling results described above, several critical limitations warrant careful consideration before widespread clinical application in CRC. First, most CRISPR knockout screens have been performed in acute leukemia models or under standard tissue culture conditions, which poorly recapitulate the immunosuppressive CRC TME. Knockout targets that enhance CAR−T function *in vitro* may not translate to the same benefit in the complex MSS CRC TME. Second, multiplex editing-knocking out three or more genes-carries cumulative genotoxicity risks, including chromosomal rearrangements, p53 pathway activation, and potential oncogenic transformation; long−term safety data from multiplex−edited CAR−T cells in CRC models are still absent. Third, while site−specific knock−in at the TRAC locus achieves uniform CAR expression and reduces tonic signaling, the efficiency of homology−directed repair in primary human T cells remains low (typically 10−30%), and the required extended ex vivo culture itself drives T cell differentiation and exhaustion. Fourth, universal CAR−T (UCAR−T) generated by TCR/B2M double knockout eliminates GVHD risk but renders cells susceptible to NK cell−mediated clearanc. Finally, the field lacks standardized metrics for comparing off−target effects and genotoxicity across different CRISPR delivery methods. Future research should prioritize head−to−head benchmarking of minimal essential edits (rather than maximal knockout numbers) combined with safer, transient delivery modalities, alongside long−term clonal tracking to assess transformation risk in CRC models.

CRISPR−based modifications enhance persistence (exhaustion knockout), safety (suicide genes, UCAR−T to avoid GVHD), and manufacturability (off−the−shelf platforms).

## 3 Preclinical and clinical research progress in CAR-T cell therapy for colorectal cancer

CAR-T clinical trials conducted for colorectal cancer cover solid tumor-specific targets and combination therapy strategies. In a phase I dose-escalation clinical trial for CEA-positive metastatic colorectal cancer, investigators demonstrated that CEA-CAR-T therapy was well tolerated in 10 patients (at doses of 1×10^5^to 1× 10^8^CAR^+^/kg), with no serious adverse events, seven patients with previous progression achieved disease stabilization, with some patients experiencing imaging tumor shrinkage and a significant decrease in serum CEA levels. CAR-T cell persistence in the peripheral blood of the high-dose group and proliferation after the second infusion were detected, suggesting clinical translational potential (17). However, recent clinical studies have reported cases of severe colitis uncontrollable by glucocorticoids combined with vedolizumab triggered by back infusion of CAR-T cells targeting CEA, highlighting the significant risk that this therapy may induce refractory gastrointestinal toxicity (58).

A non-randomized phase I clinical trial of 15 patients with refractory metastatic colorectal cancer showed that CAR-T cell therapy targeting guanylate cyclase C (GCC19) demonstrated anti-tumor activity with a controlled safety profile: an objective response rate of 40% (6/15), a median progression-free survival of 6.0 months in the high-dose group, and a median overall survival of 22.8 months, confirming the potential of CAR- T therapy to induce objective clinical responses in solid tumors (59).

A phase I clinical trial in GD2-positive solid tumors showed a favorable safety profile for third-generation GD2-specific CAR-T cell monotherapy in patients with no dose-limiting toxicities or serious adverse events. no dose-limiting toxicities or serious adverse events. However, clinical efficacy was limited despite observation of CAR-T cell immunoreactivity and myeloid cell subpopulation expansion (three out of four patients survived less than 3 months after CAR-T infusion). This study validated for the first time the feasibility of preparing CAR-T products at the bedside for solid tumors and enhanced cellular immunophenotype and expansion capacity by improving the manufacturing process (60).

A clinical study of first-generation CAR-T cells targeting tumor-associated glycoprotein (TAG)-72 (CART72) in CRC patients confirmed relative safety, but no significant anti-tumor efficacy was observed due to lack of cellular persistence and an anti-CAR immune response, suggesting that CAR constructs need to be optimized through introduction of co-stimulatory domains and fully humanized design (61).

The vascular disrupting agent CA4P significantly enhances CAR-T cell infiltration into solid tumors (elevated IFN−γ levels), and combination with CA4P dramatically enhanced CAR-T anti−tumor efficacy in subcutaneous xenograft and PDX models of human ovarian and colon cancers (62).

A phase I clinical trial in advanced CD133-positive metastatic cancer, including two CRC patients, first demonstrated that CD133-targeted CAR-T cell therapy (CART-133) showed manageable self−limiting toxicity (mainly short−term hematological toxicity). Among 23 patients, 3 (13.0%) achieved partial remission and 14 (60.9%) had stable disease; the overall 3−month disease control rate was 65.2%, with a median PFS of 5 months, and repeated infusions may prolong disease stability (27).

## 4 The immunosuppressive tumor microenvironment and clinical hurdles in CRC

Microsatellite-stable colorectal cancer makes up roughly 85% of all diagnosed cases, standing as the primary stumbling block that severely compromises the therapeutic effect of immunotherapy. Different from MSI-H colorectal cancer with high mutation load and abundant intertumoral immune cell infiltration, MSS CRC develops a typical immune-cold microenvironment and hardly responds to conventional CAR-T treatment. In this malignant microenvironment, cancer-associated fibroblasts gain persistent activation and proliferate vigorously, continuously secreting massive collagen, fibronectin and other extracellular matrix substances. These substances accumulate massively around tumor tissues and give rise to obvious stromal exclusion effect, building dense and rigid physical barriers that effectively block the directional infiltration and migration of CAR-T cells, making it difficult for effector cells to establish effective contact with target tumor cells (1).

The TGF-β signaling pathway remains abnormally hyperactive throughout tumor progression, which not only further fuels the activation of cancer-associated fibroblasts and exacerbates stromal fibrosis, but also directly interferes with the expression of intracellular cytotoxic functional molecules in CAR-T cells. It profoundly mediates immune exclusion response, induces irreversible exhaustion of infiltrated immune cells, and greatly shortens the survival cycle and anti-tumor persistence of adoptively infused CAR-T cells (63). Long-term insufficient blood perfusion leads to widespread hypoxia inside tumor lesions, and hypoxic microenvironment triggers abnormal reprogramming of tumor cell metabolism. Elevated HIF-1α level promotes the production and accumulation of adenosine, while vigorous aerobic glycolysis results in massive lactic acid deposition. Both metabolic products jointly inhibit immune cell proliferation, cytokine secretion and cytotoxic performance, and even trigger immune cell apoptosis, forming powerful metabolic immune suppression (2). Driven by sustained fibroblast activity, continuous collagen crosslinking sharply raises extracellular matrix stiffness and aggravates tissue fibrosis degree. The changed mechanical properties activate downstream YAP/TAZ mechanosignaling cascades, upregulate the expression of multiple immune checkpoint inhibitory molecules, damage the formation of stable immune synapses, and further weaken the killing capacity of CAR-T cells (64). Multiple unfavorable factors including stromal exclusion, TGF-β mediated immune inhibition, hypoxic metabolic disturbance and rigid matrix mechanical restriction interact and influence one another, jointly shaping the inherent immune-cold biological properties of MSS colorectal cancer, and posing tough clinical challenges to the popularization and optimization of CAR-T cell therapy in colorectal cancer treatment.

Collectively, these interconnected features-CAFs, TGF-β, hypoxia, adenosine, ECM stiffness, and antigen heterogeneity-explain why MSS CRC remains profoundly immune-cold and resistant to conventional CAR-T therapy. The innovative engineering strategies developed to address these obstacles (knockout of PD-1/TGFBR2, secretion of anti-PD-1/IL-12, dual-target logic-gated CARs, universal CAR-T via CRISPR, and combination with oncolytic viruses or radiotherapy) have been detailed in Section 2. Future directions building upon these advances are discussed in Section 5.

## 5 Future perspectives

Looking forward, the core direction of CRC CAR−T therapy is individualized precision intervention based on molecular typing (e.g., CMS subtypes, MSI status). Universal CAR−T (UCAR−T), generated by CRISPR−mediated knockout of TCR/HLA−I genes, can significantly shorten the preparation cycle and reduce GVHD risk, offering a true “off−the−shelf” option (65). Meanwhile, multicellular therapies such as CAR−NK and CAR−macrophage (CAR−M) are emerging as important trends. CAR−NK provides low immunogenicity, while CAR−M can degrade dense stroma and phagocytose tumor cells; both have demonstrated synergistic potential in refractory microsatellite−stable (MSS) CRC, providing new strategies to overcome drug resistance (66).

In contrast to descriptive reviews that catalog engineering platforms, this outlook directly ties each emerging strategy-molecular typing, universal CAR-T, multicellular platforms-to the physical, metabolic, and signaling barriers of the MSS CRC microenvironment characterized in Section 4. Ultimately, future breakthroughs will depend on deep cross−fertilization among oncology, immunology, and bioengineering. Oncologists must precisely define clinical needs and molecular typing, immunologists should dissect the dynamic interplay within the TME, and bioengineers need to develop modular, tunable, and controllable next−generation CAR chassis systems. Unlike previous reviews that focus solely on engineering platforms or target lists, our work systematically links next−generation designs to CRC−specific barriers and functional themes, offering a clinically oriented resource for researchers and clinicians. Only through such close interdisciplinary collaboration-coupled with continuous efforts to improve technology and treatment accessibility-can CAR−T therapy evolve from an experimental option for advanced CRC into an integral component of comprehensive treatment.

## 6 Conclusion

Chimeric antigen receptor (CAR)-T cell therapy has shown great potential in hematological malignancies, but its clinical translation in colorectal cancer (CRC) is limited by three core barriers: antigen heterogeneity, off-target toxicity, and the balance between efficacy and safety. This review summarizes the advances in next-generation CAR-T engineering tailored for CRC.

Notable progress has been made in four key areas: CAR targets have shifted to tissue-specific molecules (e.g., GUCY2C, CDH17) to reduce off-target effects; architectural optimization (armored CARs, signaling-modified constructs) enhancesanti-tumor efficacy; logic-gated circuits improve target specificity; and CRISPR-mediated gene editing enables safer, more efficient CAR-T cells.

However, the immunosuppressive tumor microenvironment (dense stroma, inhibitory cells, metabolic disorders) remains the main obstacle, limiting CAR-T persistence and efficacy. Clinical trials show preliminary anti-tumor activity but face challenges in MSS CRC and severe toxicities. Future research should focus on precision intervention based on molecular typing, universal CAR-T development, multicellular therapeutic platforms, and combined strategies to overcome TME barriers. With continuous engineering innovation and rigorous clinical validation, CAR-T therapy will become an integral part of advanced CRC treatment, improving patient outcomes.
